# Bv8-Like Toxin from the Frog Venom of *Amolops jingdongensis* Promotes Wound Healing via the Interleukin-1 Signaling Pathway

**DOI:** 10.3390/toxins12010015

**Published:** 2019-12-29

**Authors:** Jiajia Chang, Xiaoqin He, Jingmei Hu, Peter Muiruri Kamau, Ren Lai, Dingqi Rao, Lei Luo

**Affiliations:** 1 College of Life Sciences, Anhui Normal University, Wuhu 241000, China; 2 Key Laboratory of Animal Models and Human Disease Mechanisms of Chinese Academy of Sciences/Key Laboratory of Bioactive Peptides of Yunnan Province, National & Local Joint Engineering Laboratory of Natural Peptides, Kunming Institute of Zoology, Kunming 650223, China; 3 College of Life Sciences, Nanjing Agricultural University, Nanjing 210095, China; 4 Key Laboratory of National Health and Family Planning Commission on Parasitic Disease Control and Prevention, Jiangsu Provincial Key Laboratory on Parasite and Vector Control Technology, Jiangsu Institute of Parasitic Diseases, Wuxi 214064, China; 5 University of Chinese Academy of Sciences, Beijing 100049, China; 6 Sino-African Joint Research Center, Kunming Institute of Zoology, Chinese Academy of Sciences, Kunming 650223, China; 7 KIZ-CUHK Joint Laboratory of Bioresources and Molecular Research in Common Diseases, Kunming Institute of Zoology, Chinese Academy of Sciences, Kunming 650223, China; 8 Institute for Drug Discovery and Development, Chinese Academy of Sciences, Shanghai 201203, China; 9 Center for Biosafety Mega-Science, Chinese Academy of Sciences, Wuhan 430207, China; 10 State Key Laboratory of Genetic Resources and Evolution, Kunming Institute of Zoology, Chinese Academy of Sciences, Kunming 650223, China

**Keywords:** Bv8, *Amolops jingdongensis*, wound healing, Interleukin-1, keratinocytes, fibroblasts

## Abstract

Prokineticins are highly conserved small peptides family expressed in all vertebrates, which contain a wide spectrum of functions. In this study, a prokineticin homolog (Bv8-AJ) isolated from the venom of frog *Amolops jingdongensis* was fully characterized. Bv8-AJ accelerated full-thickness wounds healing of mice model by promoting the initiation and the termination of inflammatory phase. Moreover, Bv8-AJ exerted strong proliferative effect on fibroblasts and keratinocytes isolated from newborn mice by activating interleukin (IL)-1 production. Our findings indicate that Bv8 is a potent wound healing regulator and may reveal the mechanism of rapid wound-healing in amphibian skins.

## 1. Introduction

Bv8 is a family of small peptides secreted by frogs’ skin and also homologous with mammalian prokineticins, widely expressed in mammals, fish, reptiles and amphibians [1]. The precursors of Bv8 consist of a signal peptide and a highly conserved mature peptide (about 8 kDa). Its mature peptides have a conserved structural motif of five disulfide bonds formed by ten cysteine residues and a complete conservation of the N-terminal sequence of AVITGA, which is crucial for biological activities [2]. Bv8s have been identified with several regulatory functions in gastrointestinal motility, circadian rhythms, neurogenesis, angiogenesis, reproductive system, pain perception and hematopoiesis [3]. The first amphibian Bv8 was identified from *Bombina variegate* and was reported to exert contractile function on guinea pig ileum and hyperalgesia function in rats [4]. In this study, we reveal a Bv8-like peptide, Bv8-AJ, with significant function acting as a potent wound healing regulator.

For wound healing to take place, a sophisticated interaction of resident epithelial cells mainly keratinocytes (KC), fibroblasts (FB) and recruited kinds of inflammatory cells occur to enhance the three stages of wound repair: an inflammatory stage, a proliferative stage, and a remodeling stage [5]. Full-thickness wound in mice model demonstrated that Bv8-AJ exerted strong activity in accelerating wound closure. Pathological sections were exploited to understand the influence of Bv8-AJ on the three phases of wound healing. To further investigate the mechanisms of Bv8-AJ in accelerating wound healing, the effect of Bv8-AJ on cell proliferation, IL-1 production, and mitogen-activated protein kinase (MAPK) signaling pathway were evaluated. 

## 2. Results

### 2.1. Bv8-AJ Purification 

**Figure 1:**
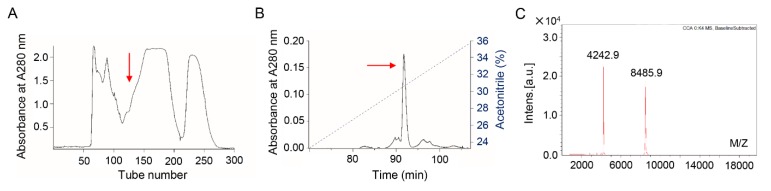
Purification of Bv8-AJ from the skin secretions of *A. jingdongensis*. (**A**) Sephadex G-50 gel filtration of skin secretions of *A. jingdongensis*. The fraction with contractile activity on isolated rat ileum is marked by an arrow. (**B**) The fraction with contractile activity on isolated rat ileum from the Sephadex G-50 gel filtration was further purified by C18 RP-HPLC column. The elution was performed with the indicated gradient of acetonitrile at a flow rate of 1.0 mL/min. The purified Bv8-AJ is indicated by an arrow. (**C**) MALDI-TOF MS analysis of the purified Bv8-AJ.

The supernatant of *A. jingdongensis* skin secretions was divided into seven fractions by Sephadex G-50 gel filtration as illustrated in Figure 1A. The fraction with contractile activity on isolated rat ileum (data not shown) is marked with a red arrow. The active fraction was further applied to a C8 RP-HPLC column as illustrated in Figure 1B, and the purified peptide (Bv8-AJ) is indicated by an arrow. The molecular weight of Bv8-AJ identified by MALDI-TOF mass spectrometry is 8485.9 Da (Figure 1C). 

### 2.2. Bv8-AJ Structural Characterization and Sequence Alignment

**Figure 2:**
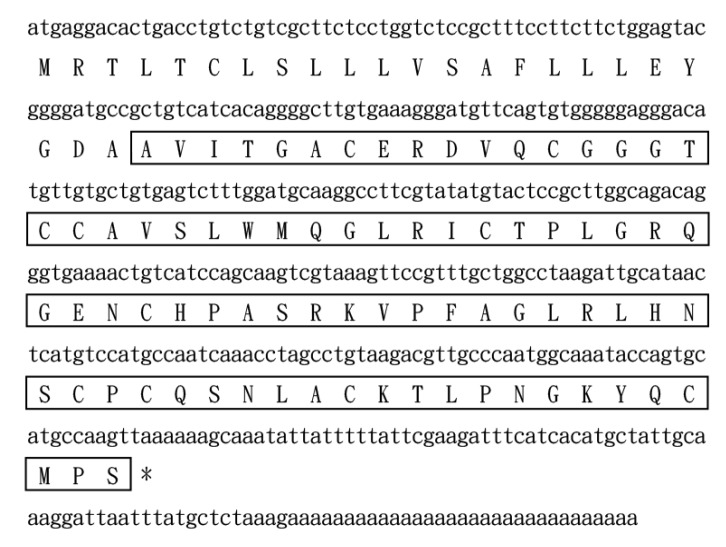
The nucleotide sequence and the deduced amino acid sequence of Bv8-AJ. The mature peptide of Bv8-AJ is boxed. The asterisk (*) indicates the stop codon.

**Figure 3:**
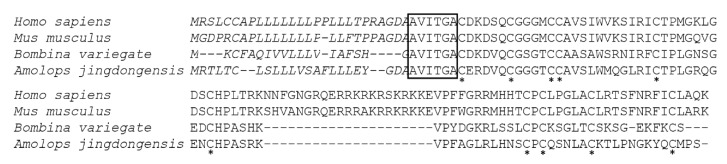
Amino acid sequence alignment of Bv8-AJ precursor with other members of prokineticins. The signal peptide is shown in italic format. The open box indicates the conserved N-terminal hexapeptide sequence (AVITGA) of mature peptide. Identical cysteines in mature peptide are indicated by an asterisk (*). The gaps (-) were introduced for optimal comparison.

Bv8-AJ amino acid sequence was preliminarily determined as AVITGACERDVQCGGGTCCA VSLWMQGLRICTPLGRQG by Edman degradation. In order to obtain the complete sequence of Bv8-AJ, the cDNA library of *A. jingdongensis* frog skin was constructed. Upon screening of the skin cDNA library, several clones containing inserts of around 450 base pairs were isolated and sequenced. The complete nucleotide sequence (GenBank accession number: KC997226) and the deduced amino acid sequence of Bv8-AJ are shown in Figure 2. Bv8-AJ contains a coding region of 309 nucleotides, and the coding precursor corresponds to a polypeptide of 103 amino acids including a signal peptide and a mature peptide. The mature peptide was composed of 80 amino acid residues including 10 cysteines, which form 5 intra-molecular disulfide bridges as described previously [6]. BLAST search indicated Bv8-AJ is a member of prokineticin superfamily containing a conserved N-terminal sequence (AVITGA), the same structural motifs and cysteine distribution pattern with other members (Figure 3).

### 2.3. Bv8-AJ Accelerated Full-thickness Wound Healing in Mice

**Figure 4:**
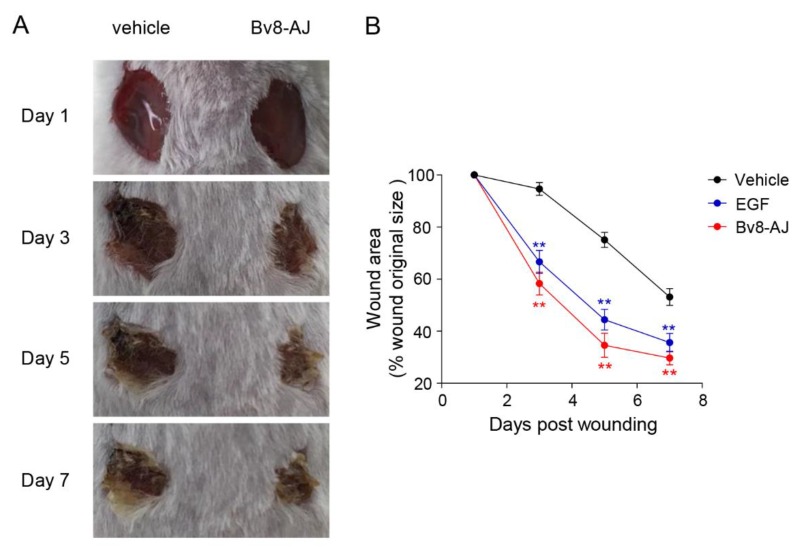
Effects of Bv8-AJ on full-thickness wounds in mice. (**A**) Representative photographs. (**B**) The percentage of wound area at days 3, 5 and 7 to day 1 after treatment with vehicle, Bv8-AJ and EGF, respectively. Data represent mean values ± S.E.M. of three mice (Vehicle, n = 6; EGF, n = 3; Bv8-AJ, n = 3). Bv8-AJ, 2 (2.26 × 10^−^^4^ µM) μg/day/wound. EGF, 2 (3.31 × 10^−^^4^ μM) μg/day/wound. ** *p* < 0.01, significant difference compared to the original wound size.

The effects of Bv8-AJ on full-thickness wound in mice are shown in Figure 4. Bv8-AJ accelerated the wound closure in a time-dependent manner. At day 3, 5 and 7, the percentage of wound areas to day 1 were 58.3%, 34.6% and 29.7% for Bv8-AJ treatment group, respectively, while the percentages were 95.6%, 73.7% and 50.9% for vehicle group. At the same mass concentration (2 μg/day/wound), the effect of Bv8-AJ (2.26 × 10^−4^ µM/day/wound) on wound healing was more remarkable than that of EGF (3.31 × 10^−4^ μM/day/wound). The percentage of day 3, 5 and 7 to day 1 for EGF treatment just reached up to 66.7%, 44.3% and 35.6%, respectively.

**Figure 5:**
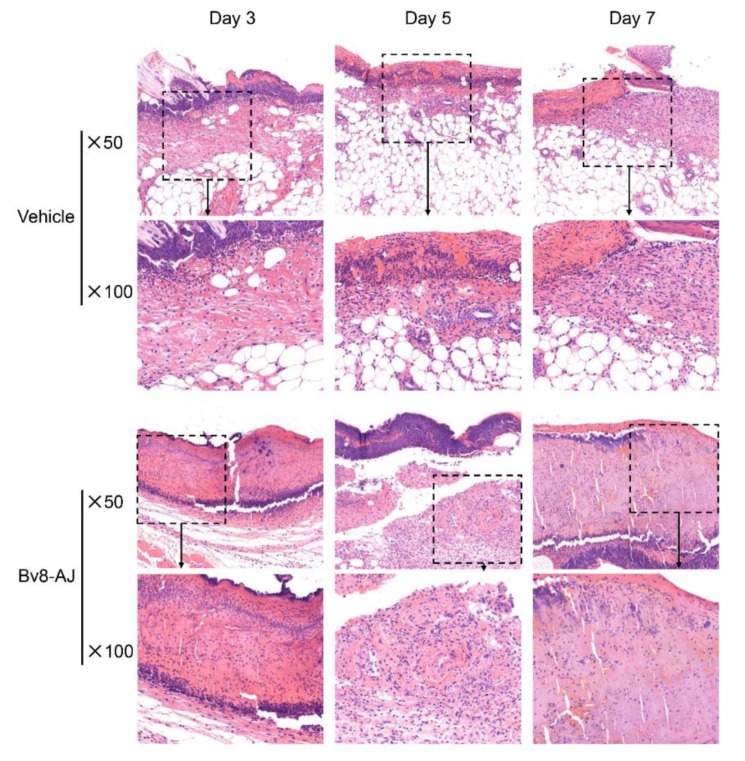

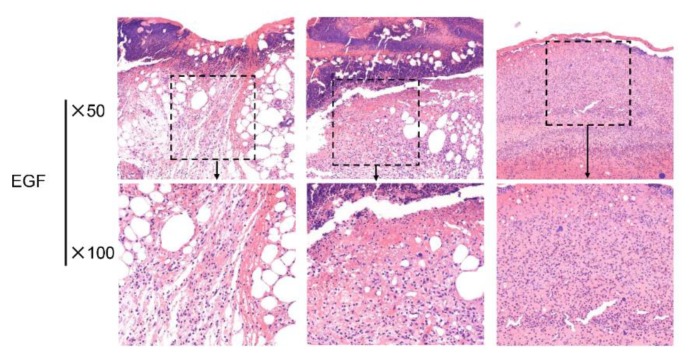
Representative pathological sections of full-thickness wounds in mice at day 3, day 5 and day 7 after treatment of vehicle, Bv8-AJ and EGF, respectively. H&E staining, magnification 50× and 100× as indicated.

The effect of Bv8-AJ on wound healing was further evaluated through histopathological study as shown in Figure 5. Bv8-AJ accelerated the initiation and the end of inflammatory phase of wound healing. 

Compared to vehicle-treated group, pathological sections of Bv8-AJ-treated group showed a slightly increased inflammatory cell (small blue dots) infiltration at day 3 and a significantly reduced inflammatory cell infiltration at day 7. This also showed a reduction in epidermis thickness and scar depth on day 7 after Bv8-AJ treatment.

### 2.4. Bv8-AJ Promoted Cell Proliferation

**Figure 6:**
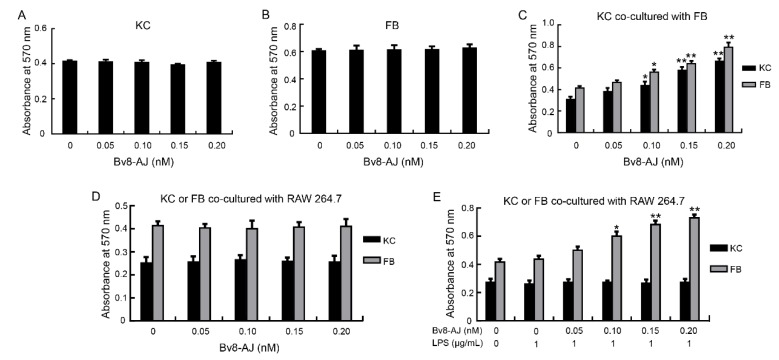
Effects of Bv8-AJ on cell proliferation. (**A**) Effect of Bv8-AJ on KC proliferation when KC was cultured alone. (**B**) Effect of Bv8-AJ on FB proliferation when FB was cultured alone. (**C**) Effects of Bv8-AJ on KC and FB proliferations when they were cultured together. (**D**) Effects of Bv8-AJ on KC or FB proliferation when KC or FB was co-cultured with RAW 264.7, respectively. (**E**) Effects of Bv8-AJ on KC or FB proliferation when KC or FB was co-cultured with 1 μg/mL of LPS-activated RAW 264.7, respectively. Cells were treated with different concentrations of Bv8-AJ as indicated. Data represent mean values ± S.E.M. of three independent experiments in duplicates. * *p* < 0.05, ** *p* < 0.01, significant difference compared to the group without treatment of Bv8-AJ.

The effects of Bv8-AJ on cell proliferation are shown in Figure 6. Bv8-AJ had no direct proliferative effects on keratinocytes (KC) and fibroblasts (FB) (Figure 6A,B). Interestingly, Bv8-AJ promoted KC and FB proliferation in a dose-dependent manner when they were cultured together. At concentrations of 0.05, 0.10, 0.15 and 0.20 nM, Bv8-AJ increased the percentage of FB proliferation by 12.9%, 35.4%, 54.9% and 91.9%, while KC proliferation was increased by 23.6%, 41.9%, 88.1% and 115.0%, respectively (Figure 6C). 

The proliferative effects of Bv8-AJ on KC or FB co-cultured with RAW 264.7 were also investigated. Bv8-AJ had no effects on their proliferation when KC or FB were co-cultured with RAW 264.7 (Figure 6D). However, Bv8-AJ promoted FB proliferation in a dose-dependent manner when FB co-cultured with 1 μg/mL of LPS-activated RAW 264.7 (Figure 6E). At the concentrations of 0.05, 0.10, 0.15 and 0.20 nM, Bv8-AJ increased FB proliferation up to 14.5%, 37.4%, 56.5%, 67.2% compared to LPS (1 μg/mL)-treatment alone. 

### 2.5. Bv8-AJ Induced IL-1 Production 

**Figure 7:**
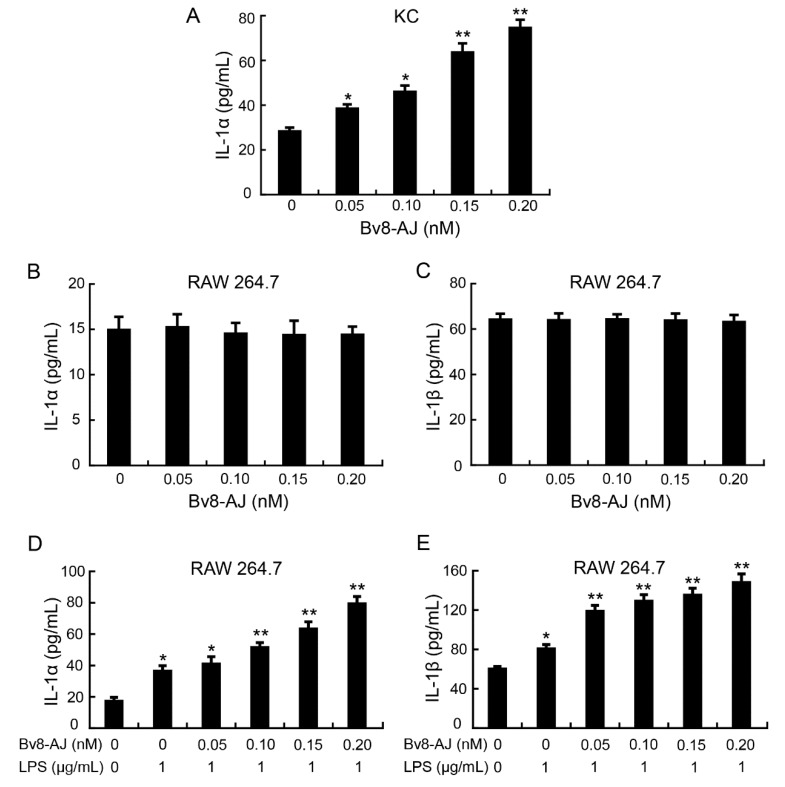
Effects of Bv8-AJ on IL-1 production. (**A**) Effect of Bv8-AJ on IL-1α production in KC. (**B**) Effect of Bv8-AJ on IL-1α production in RAW 264.7. (**C**) Effect of Bv8-AJ on IL-1β production in RAW 264.7. (**D**) Effect of Bv8-AJ on IL-1α production in 1 μg/mL of LPS-activated RAW 264.7. (**E**) Effect of Bv8-AJ on IL-1β production in 1 μg/mL of LPS-activated RAW 264.7. Cells were treated with different concentrations of Bv8-AJ as indicated. Data represent mean values ± S.E.M. of three independent experiments in duplicates. * *p* < 0.05, ** *p* < 0.01, significant difference compared to the group without treatment of Bv8-AJ and LPS.

The effect of Bv8-AJ on the production of IL-1 in KC, FB and RAW 264.7 are illustrated in Figure 7. Bv8-AJ induced the secretion of IL-1α in KC in a dose-dependent manner. 

At concentrations of 0.05, 0.10, 0.15 and 0.20 nM, Bv8-AJ induced 36.8%, 63.3%, 126.7% and 166.1% increase of IL-1α production, respectively (Figure 7A). The levels of IL-1β in Bv8-AJ-treated KC, IL-1α and IL-1β in Bv8-AJ-treated FB were undetectable. Bv8-AJ had no effect on the production of IL-1α and IL-1β in RAW 264.7 cells (Figure 7B,C), while it promoted IL-1α and IL-1β production dose-dependently at 1 μg/mL LPS-activated RAW 264.7. At concentrations of 0.05, 0.10, 0.15 and 0.20 nM, Bv8-AJ increased 12.5%, 41.4%, 74.0%, 118.2% IL-1α production (Figure 7D) and 47.4%, 60.2%, 67.9%, 83.8% IL-1β production (Figure 7E) in RAW 264.7 cells at the presence of LPS (1 μg/mL) compared to LPS (1 μg/mL)-treatment alone, respectively. There were no significant effects of Bv8-AJ on other cytokines’ production (data not shown).

### 2.6. IL-1 Receptor Antagonist Inhibited Bv8-AJ-induced Cell Proliferation

**Figure 8:**
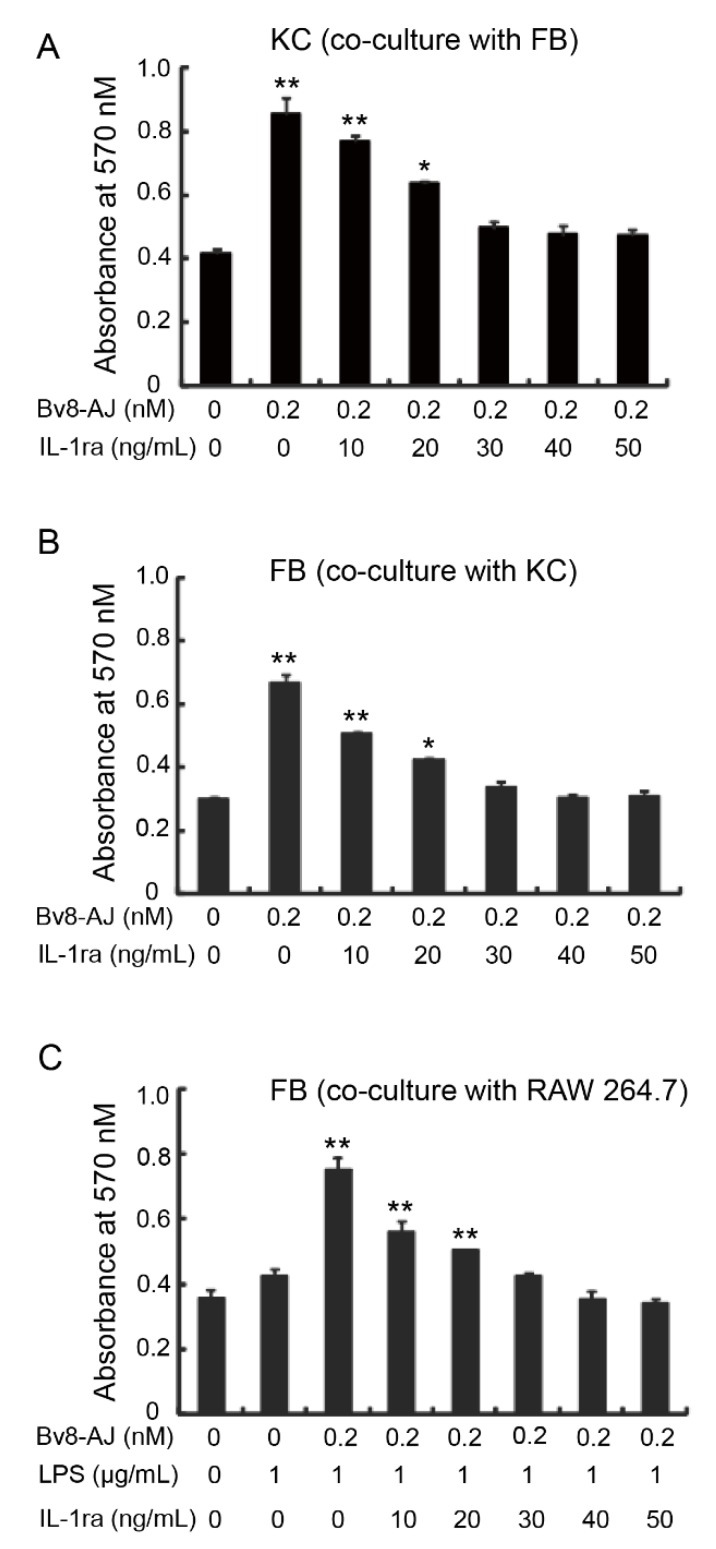
Inhibitory effects of IL-1ra on Bv8-AJ-induced cell proliferation. (**A**) Inhibitory effect of IL-1ra on proliferation of KC cells induced by Bv8-AJ in co-culture with FB. (**B**) Inhibitory effect of IL-1ra on proliferation of FB cells induced by Bv8-AJ in co-culture with KC. (**C**) Inhibitory effect of IL-1ra on Bv8-AJ-induced cell proliferation in FB when it was co-cultured with 1 μg/mL of LPS-activated RAW 264.7. Cells were treated with Bv8-AJ and IL-1ra at different concentrations as indicated. Data represent mean ± S.E.M. * *p* < 0.05, ** *p* < 0.01, significant difference compared to the group treatment of 0.2 nM of Bv8-AJ and 0 ng of IL-1ra.

The effects of IL-1 receptor antagonist (IL-1ra) on Bv8-AJ-induced cell proliferation are shown in Figure 8. 0.2 nM of Bv8-AJ significantly induced KC and FB proliferations when they were cultured together. However, the proliferative effects of Bv8-AJ on KC and FB were inhibited by IL-1ra in a dose-dependent manner, and were completely inhibited by 40 ng/mL of IL-1ra (Figure 8A,B). IL-1ra also inhibited Bv8-AJ-induced (0.2 nM) FB proliferation when it co-cultured with 1 μg/mL of LPS-activated RAW 264.7, and proliferative effect was completely inhibited by 40 ng/mL IL-1ra.

### 2.7. Bv8-AJ activated MAPK Signaling Pathway in KC and RAW 264.7 Cells

**Figure 9:**
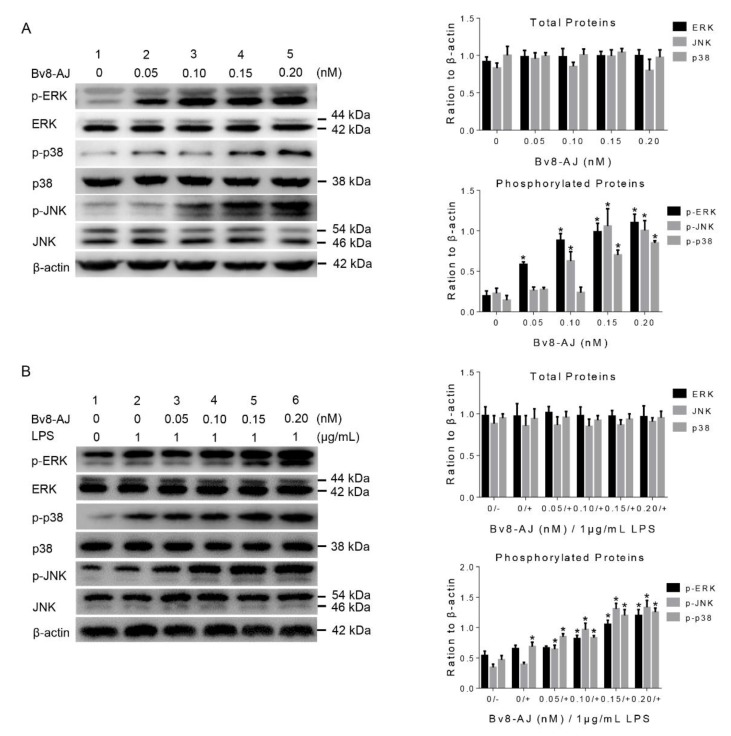
Effects of Bv8-AJ on MAPK signaling pathway by western blot analyses. (**A**) Effects of Bv8-AJ on MAPK signaling pathway in KC. (**B**) Effects of Bv8-AJ on MAPK signaling pathway in 1 μg/mL of LPS-activated RAW 264.7. All the bars represent mean ± S.E.M. Significant statistics difference indicated by asterisks (* *p* < 0.05).

To investigate the mechanisms of IL-1 production, the effects of Bv8-AJ on MAPK signaling pathway in KC and RAW 264.7 cells were further exploited by western blot analyses as illustrated in Figure 9. Bv8-AJ significantly activated the phosphorylations of ERK, JNK and p38 in both KC and LPS-activated RAW 264.7 cells in a dose-dependent manner. At the concentration of 0.20 nM, Bv8-AJ increased expressions of 135.0% p-ERK1, 429.3% p-ERK2, 401.0% p-JNK1, 770.8% p-JNK2, 397.9% p-p38 in KC (Figure 9A) and 184.7% p-ERK1, 328.1% p-ERK2, 351.5% p-JNK1, 590.0% p-JNK2, 621.5% p-p38 in LPS-activated RAW 264.7 (Figure 9B).

## 3. Discussion

Amphibian skins are naked and thus exposed to harsh environmental and ecological conditions. This characteristic makes them to have extreme chemical diversity [7,8]. Till now, several biochemical arsenals with potential pharmaceutical properties, particularly antimicrobial peptides (AMPs), have been discovered from the skin secretions of amphibians [9,10,11,12,13,14]. Despite this, little information is available about amphibian skin-derived bioactive compounds with wound healing activity. The naked skins of amphibians are predisposed to numerous types of injuries such as physical damage, microbial infections, predation and other kinds of wounding [15]. These skins have therefore evolved a unique strategy to defend these forms of wounding. Hence, it is accordingly reasonable to identify wound healing mediators from the secretion of amphibian skins.

A Bv8-like peptide, Bv8-AJ, was recognized from the secretions of *A. jingdongensis* skin. Bv8-AJ belongs to the Bv8 family with a complete conservation of the first six amino acids (AVITGA) of mature peptides, the same structural motif of five disulfide bridges and their distribution patterns [2,6,16,17,18]. Bv8-AJ exhibited a strong property in accelerating wound closure, which was more effective compared to EGF (Figure 4). Further investigation indicated that Bv8-AJ induced production of IL-1α in KC and IL-1 (IL-1α and IL-1β) in LPS-activated RAW 264.7 cells dose dependently (Figure 7). IL-1 signaling is a key pathway that control KC and FB proliferation. IL-1 produced by KC and LPS-activated RAW 264.7 may activate peroxisome proliferator-activated receptor β/δ (PPARβ/δ) expression in FB, which in turn activates the expression of mitogenic factors [19]. These mitogenic factors exerted direct proliferative effects on KC and FB when they were co-cultured together. Bv8-AJ activated MAPK signaling pathway in KC (Figure 9A) and LPS-activated RAW 264.7 (Figure 9B), which in turn contributed to IL-1 production (Figure 7) and cell proliferation (Figure 6), finally accelerated the initiation and the termination of inflammatory phase.

Over recent years, a number of amphibian peptides such as AH90, βγ-CAT, Ot-WHP, CW49, and Bm-TFF2 has been discovered and they have been reported to play important roles in angiogenesis and wound healing [20,21,22,23,24]. AH90 was identified from skin secretions of frog *Odorrana grahami*. It was found that AH90 promoted release of transforming growth factor β1 (TGF-β1) through activation of nuclear factor-κB (NF-κB) and c-Jun NH2-terminal kinase (JNK) mitogen-activated protein kinases signaling pathways and showed potential wound healing-promoting activity [20]. βγ-crystallin fused aerolysin-like protein (α-subunit) and trefoil factor (β-subunit) complex (βγ-CAT) is a complex of a bacterial pore-forming toxin aerolysin-like protein and trefoil factor identified in the frog *Bombina maxima.* βγ-CAT treatment increased the expression of IL-1β, TGF-β1, vascular endothelial growth factor (VEGF) and basic fibroblast growth factor (bFGF), and accelerated the healing of full-thickness wounds [21]. Our present study reveals that Bv8-AJ, a novel Bv8-like peptide, exerted strong proliferative effect on KC and FB by activating IL-1 production via MAPK signaling pathway. Given the importance of cutaneous wound healing and the extensive cost to synthesize venom, amphibian frugally uses venom to fulfill the physiologic requirement of rapid tissue repair. Together with previous studies of the different wound healing peptides isolated from different amphibian species, our study suggests the existence of functional convergence evolution in wound healing peptides.

Bv8-AJ study has enlightened us on the wound healing mechanism of amphibian skins however, this is still far from enough. Taking advantage of advanced molecular approaches, such as transcriptomics, proteomics and metabolomics, we may have a more comprehensive and systematic understanding on amphibian skin wound healing modulators, targets and related signaling pathways. In other words, the research revealing the mechanism of rapid wound-healing in amphibian skins is still on the way. The discovery of Bv8-AJ may help us to better understand the rapid wound-healing tactics of amphibians, though much more wound healing-related molecules need to be further explored. Given that animal toxins are good resources for pharmacological research and clinic therapeutics, the results from our study may help us to reveal the wound healing mechanism of amphibian skins and lay a ground work for the development of new drugs in wound healing.

## 4. Conclusions

In conclusion, a Bv8-like peptide, Bv8-AJ, was identified from the skin of *A. jingdongensis*. In mice model, Bv8-AJ potentially accelerated wound healing by speeding up the initiation and the end of inflammatory phase, as well as proliferative phase. In addition, Bv8-AJ exerted strong proliferative effects on FB and KC by activating IL-1 signaling pathway. The current work identified that Bv8 is a potent wound healing regulator and may reveal the mechanism of rapid wound-healing in amphibian skins.

## 5. Materials and Methods

### 5.1. Animals

Kunming mice (aged 2–3 week, 18–23 g, both sexes) were purchased from the SPF Laboratory Animal Center of Kunming Institute of Zoology (KIZ). *A. jingdongensis* frogs (adult, body weight range 25–35 g, both sexes, n = 30) were collected from Jingdong Yi Autonomous County, Yunnan, China. SD Rats (aged 6–8 week, 200–300g, both sexes) were purchased from Laboratory Animal Research Center of Kunming Medical University. All the experimental schemes were permitted by the Institutional Animal Care and Use Committees of Kunming Institute of Zoology, Chinese Academy of Sciences (identification code: SMKX-2011136; date of approval: 12 December 2011). Efforts were made to minimize the number of animals used and unnecessary suffering.

### 5.2. Collection of Frog Skin Secretions

*A. jingdongensis* skin secretions were gathered as previously described [25,26]. Briefly, we used the volatile anhydrous ether soaked in absorbent cotton to stimulate the skin of frogs and the secretions were seen to exude. Frog skins were washed with 0.1 M PBS (pH 7.0) and a total volume of 1000 mL of skin secretion solutions were collected and centrifuged at 4 °C, 13,000 rpm for 10 min. The skin secretion supernatants were filtrated with disposable needle filter (SLGP033RB, Millipore, Merck, Darmstadt, Germany) and lyophilized for further use.

### 5.3. Peptide Purification

About 6.0 g of lyophilized skin secretions of *A. jingdongensis* was used for further purification. 0.5g of freeze-dried skin secretion was dissolved in 5 mL PBS (0.1 M, pH 7.0) and centrifuge at 4 °C, 13,000 rpm for 10 min. The supernatant (about 5 mL) was gently loaded into a Sephadex G-50 (2.6 cm × 100 cm, GE Healthcare Life Sciences, Marlborough, MA, USA) gel filtration column equilibrated with PBS (0.1 M, pH 7.0). Elution was performed with the same PBS buffer and fractions were gathered every 10 min with a speed of 0.3 mL/min. The eluate was monitored with absorbance of 215 nm/280 nm by spectrophotometer (Alpha-1900Splus, LASPEC, Shanghai, China). The contractile activity of seven fractions purified by gel filtration on isolated rat ileum was measured as described previously [27]. Briefly, after euthanizing rats under anesthesia, the distal ileum was removed immediately and immersed in oxygen-filled with Tyrode’s solution (Solarbio, Beijing, China). About 2 cm segments of the isolated ileum cutting section were installed in a 5 mL bath containing Tyrode’s solution, which was kept at 37 °C and charged with oxygen. BL-420L software package (TECHMAN, Chengdu, China) was used for the collection and analysis of contractile activity. Bradykinin was used as positive control [28]. The fractions with contractile activity were pooled and further purified by a C_8_ RP-HPLC (XBridge^TM^, C8, 5 μm, 25 × 0.46 cm, Waters, Milford, MA, USA). Elution was performed under solution A (water, 0.1% trifluoroacetic acid, *v*/*v*) and solution B (acetonitrile, 0.1% trifluoroacetic acid, *v*/*v*) with a linear solution B gradient of 0-100% as described previously [29]. During the elution, the absorbance value of the eluate was monitored at 215 nm/280 nm. The UV-absorbing peaks were gathered, lyophilized, and assayed for contractile activity.

### 5.4. Structural Analysis

1.0 µL of the purified peptide collected from RP-HPLC was detected and analyzed on a MALDI-TOF mass spectrometer (Bruker Daltonik GmbH, Leipzig, Germany) for molecular weight detection and purity confirmation. Upon confirmation, partial amino acid sequences were revealed by Edman degradation analysis on an Applied Biosystems pulsed liquid-phase sequencer (ABI 491, Carlsbad, CA, USA).

### 5.5. cDNA Cloning

SMART^TM^ techniques were used to synthesize the cDNA according to previous methods [30]. RNeasy Protect Mini Kit (QIAGEN, Hilden, Germany) was used to extracted total RNA of *A. jingdongensis* frog skin [12]. Following the manufacturer’s instructions, *A. jingdongensis* frog skin cDNA library was constructed containing about 3.3 × 10^5^ independent colonies. PCR method was then used to screen and clone the cDNA library. Two oligonucleotide primers, 5’ PCR primer (5’-AAGCAGTGGTATCAACGCAGAGT-3’) and specific primer designed according to partial amino acids of Bv8-AJ (5’-A(C/T)TG(A/C/G/T)AC(A/G)TC(A/C/G/T)C(G/T)(C/T)TC(A/G)CA(A/C/G/T)GC-3’), were used in PCR reaction. The full length cDNA sequence was ultimately cloned from the cDNA library using the S1 primer (5’-ATGAGGACACTGACCTCTG-3’, sense primer) and 3’ PCR primer (antisense primer) provided in the library kit. All PCR conditions were 95 °C (4 min), followed by 28 cycles of 95 °C (30 s), 52 °C (30 s), 72 °C (40 s), and extended by 10 min to 72 °C. DNA sequencing was conducted on ABI 3730XL DNA sequencer (ABI, Carlsbad, CA, USA).

### 5.6. Effects of Bv8-AJ on Full-thickness Wounds in Mice

Mice were randomly divided into groups and placed separately in ventilated cages to prevent fighting and licking each other or any other possible means of contact. After mice were anesthetized through intraperitoneal injection of sodium pentobarbital (1%, *w*/*v*, 100 μL per mouse), two full-thickness wounds were made on both back sides of each mouse’s back. Wounds were treated with 20 µL of vehicle (0.9% saline), Bv8-AJ (100 µg/mL) or recombinant murine EGF (100 μg/mL, PeproTech Inc, Rocky Hill, NJ, USA, positive control) from the first day to the seventh day (day 1–7). On day 3, 5 and 7 post-wounding, mice were euthanized and the wounded skin tissues were taken and fixed in 10% formalin (Sigma-Aldrich, St. Louis, MO, USA) in PBS for 24 h. After fixation, the tissues were dehydrated by increasing concentrations of alcohol in turns, embedded in paraffin and sliced to a thickness of 5 µm using histocut (RM2235, Leica, Wetzlar, Germany). Hematoxylin and eosin stained sections were observed by microscope (BX51, Olympus, Tokyo, Japan).

### 5.7. Isolation of Fibroblasts and Keratinocytes from Newborn Mice

FB and KC were separated from skins of nascent mice as previously mentioned [31]. Briefly, the newborn Kunming mice were executed with deep CO_2_ anesthesia and sterilized. The skin were quickly removed, expanded, and floated on a cryogenic trypsin (with not EDTA, 0.25%, *w*/*v*, Sigma-Aldrich, Shanghai, China) overnight with the dermis side-down at 4 °C. The skins were treated with typsin, and then transferred to dry plates with the epidermis facing down and in contact with the plastic at various points on the edges. The dermis was directly raised higher than the epidermis. To prepare FB cells, dermis was transferred to plates containing Dulbecco’s Modified Eagle Media (DMEM, Solarbio) with 10% FBS, penicillin (200 U/mL), and streptomycin (200 μg/mL, Invitrogen, Carlsbad, CA, USA). To prepare KC cells, epidermis was transferred to plates containing S-MEM (Solarbio) chelexed with chelex®100 Resin (Bio-Rad, Hercules, CA, USA), 8% FBS, penicillin (200 U/mL) and streptomycin (200 μg/mL). After fully mincing and triturating, the suspension was filtered using cell strainer (1250 mesh, BD, Franklin Lakes, NJ, USA). After incubation for 24 h, the isolated cells were washed with PBS solution, while the suspension cells and fragments were discarded. The isolated cells were incubated in 5% CO_2_ at 37 °C.

### 5.8. Cell Proliferation Assay

KC and FB were cultivated on 96-well plates with a density of 1 × 10^4^ cells/well. For the co-cultured cells, some were seeded in 24-well plates while the others were seeded in Millicell (Millipore Merck). Cells were treated with various concentrations (0, 0.05, 0.10, 0.15, 0.20 nM) of Bv8-AJ and/or LPS (0, 1 μg/mL, from *Escherichia coli* 055:B5, Sigma-Aldrich) for 24 h. At the end of incubation, 10% volume of MTT (5 mg/mL) was added and hatched for additional 4 h. Cells were then dissolved in 200 µL of dimethylsulfoxide (DMSO, Beyotime, Shanghai, China) and absorbance at 570 nm was detected by a microplate reader (Epoch Etock, BioTek, Winoski, VT, USA) [32,33]. 

### 5.9. Effects of Bv8-AJ on Cytokines Production

Cells (2 × 10^5^) were seeded on 96-well plates and incubated with different concentrations (0, 0.05, 0.10, 0.15, 0.20 nM) of Bv8-AJ and/or LPS (0, 1 µg/mL) for 48 h. Supernatants of the culture medium were harvested for IL-1α, IL-1β, TNF-α, TGF-β, IL-6 and IL-10 assays. Production levels of cytokines were determined by mouse ELISA kits (DAKAWE, Beijing, China) according to the manufacturer’s instruction.

### 5.10. Effects of IL-1ra on Bv8-AJ-induced Cell Proliferation

To investigate the relationships between cell proliferation and IL-1 production, cells were seeded, and a serial concentrations (0, 10, 20, 30, 40, 50 ng/mL) of mouse IL-1ra (R&D, Minneapolis, MN, USA) were added. Cell proliferation was measured after incubation for 24 h. All the methods used for cell culture and cell proliferation assay were the same as described above.

### 5.11. Western Blot Analysis

KC and RAW 264.7 cells (1 × 10^6^/well) were inoculated on 12-well plates, and cultivated with 3% FBS (*v*/*v*) S-MEM or DMEM medium for 16 h, respectively. KC were treated with different concentrations (0, 0.05, 0.10, 0.15, 0.20 nM) of Bv8-AJ for 24 h. RAW 264.7 cells were pretreated with a serial concentrations (0, 0.05, 0.10, 0.15, 0.20 nM) of Bv8-AJ for 24 h before the incubation of LPS (0, 1 μg/mL) for 30 min. Cells were washed three times with ice-cold PBS solution, gathered and then centrifuged at 13,000 rpm, 4 °C for 5 min. The washed pellets were resuspended with RIPA lysis buffer (Biodragon, Beijing, China) and incubated on ice for half an hour. The cells residue were then removed through centrifugation of the cell lysate at 13,000 rpm, 4 °C for 10 min, and the supernatant were stored at −80 °C for western blot analysis. BCA protein assay kit (BioTeke, Beijing, China) was used to determine protein concentration. 30 μg protein was electrophoresed to a gel concentration of 12% by polyacrylamide gel electrophoresis, and then the gel was electrically transferred to polyvinylidene fluoride (PVDF) membrane (Millipore Merck). 5% skimmed milk powder (BD) dissolved in TBST was used to block the immunoblot PVDF membrane at 25 °C for 2 h. Subsequently, primary antibody against β-actin (1:5000, Santa Cruz Biotechnology, Santa Cruz, CA, USA), ERK1/2, JNK, and p38 (1:2000; Cell Signaling Technology, Danvers, MA, USA) was added for an overnight incubation at 4 °C, respectively. After washing with TBST for 15 min/5 min each time, blots were incubated at 25 °C for 1 h with horseradish peroxidase combined with the second antibody. Blots were washed again with TBST for three times, and the signals were detected by enhanced chemiluminescence kit (TIANGEN, Beijing, China).
